# Exploratory analysis of covariation of microbiota-derived vitamin K and cognition in older adults

**DOI:** 10.1093/ajcn/nqz220

**Published:** 2019-09-13

**Authors:** Angela McCann, Ian B Jeffery, Bouchra Ouliass, Guylaine Ferland, Xueyen Fu, Sarah L Booth, Tam T T Tran, Paul W O'Toole, Eibhlís M O'Connor

**Affiliations:** 1 School of Microbiology, University College Cork, Cork, Ireland; 2 APC Microbiome Ireland, University College Cork, Cork, Ireland; 3 Département de Nutrition, Université de Montréal, Montreal, Canada; 4 Jean Mayer US Department of Agriculture Human Nutrition Research Center on Aging at Tufts University, Boston, MA, USA; 5 Department of Biological Sciences, School of Natural Sciences, University of Limerick, Limerick, Ireland; 6 Health Research Institute, University of Limerick, Limerick, Ireland

**Keywords:** microbial menaquinone biosynthesis, elderly, cognition, shotgun metagenomic sequencing, vitamin K

## Abstract

**Background:**

Vitamin K has multiple important physiological roles, including blood coagulation and beneficial effects on myelin integrity in the brain. Some intestinal microbes possess the genes to produce vitamin K in the form of menaquinone (MK). MK appears in higher concentration in tissues, such as the brain, particularly MK4, than the dietary form of phylloquinone (PK). Lower PK concentrations have been reported in patients with Alzheimer disease while higher serum PK concentrations have been positively associated with verbal episodic memory. Despite knowledge of the importance of vitamin K for various health parameters, few studies have measured MK concentration and biosynthesis by gut commensals.

**Objective:**

The aim of the current study was to investigate the relation between genes involved in gut-microbiota derived MK, concentrations of MK isoforms, and cognitive function.

**Methods:**

Shotgun metagenomic sequencing of the gut microbiome of 74 elderly individuals with different cognitive ability levels was performed. From this, gene counts for microbial MK biosynthesis were determined. Associations between clusters of individuals, grouped based on a similar presence and prevalence of MK biosynthesis genes, and cognitive ability were investigated. Fecal MK concentrations were quantified by HPLC to investigate correlations with subject clusters.

**Results:**

Separation of subject groups defined by banded quantification of the genetic potential of their microbiome to biosynthesize MK was associated with significant differences in cognitive ability [assessed using the Mini-Mental State Examination (MMSE)]. Three MK isoforms were found to be positively associated with MMSE, along with the identification of key components of the MK pathway that drive this association. Although the causality and direction of these associations remain unknown, these findings justify further studies.

**Conclusions:**

This study provides evidence that although total concentrations of MK did not covary with cognition, certain MK isoforms synthesized by the gut microbiome, particularly the longer chains, are positively associated with cognition.

## Introduction

Menaquinone (MK), also known as vitamin K_2_, forms an essential part of the electron-transfer chain in micro-organisms that use respiration to energize the cell. Dietary MK can be sourced from bacterially fermented food such as cheese and natto (1); in mammals, some gut microbes harbor genes encoding enzymes for MK production that occurs by 1 of 2 pathways (2).

MKs can be classified into 15 subtypes based on the number of isoprene units in their side chain (3). The major MK component in many Gram-positive spore-forming bacteria is MK7; in *Escherichia coli*, it is MK8 (4); and within the genus *Bacteroides*, the long-chain forms MK10 and MK11 are primarily synthesized (5).

Vitamin K also exists in another biologically active form, phylloquinone (PK; vitamin K_1_), which forms the major dietary vitamin K component in green vegetables (6). Diet-derived PK can be converted to MK4 (7) and is estimated to occur for 5–25% of digested PK (8, 9). Despite this interconversion, PK concentrations in animal tissues are remarkably low compared with MKs, particularly MK4 (10). In the liver, PK is only a minor constituent, with long-chained MK7 to MK13 making up ∼90% of the stored vitamin component (11). MKs 10, 11, and 12 are present in low amounts in a limited number of dietary sources (1) but are produced by certain gut bacteria (e.g., *Bacteroides* spp.) (5, 12, 13). MK has been reported to be more effective than PK with respect to maintaining cardiovascular health (14) and slowing atherosclerotic progression (4).

Emerging evidence supports novel roles for the influence of the gut microbiota on behavior and the brain (15, 16). Evidence is also mounting for an expanded role for vitamin K beyond coagulation (17) in processes including cognition. A link to brain function was first reported in cases of central nervous system abnormalities in infants exposed in utero to vitamin K antagonists (18). In addition, vitamin K is involved in alterations in the metabolism of sphingolipids (19), which have been linked to aging, Parkinson disease, and Alzheimer disease (20). In addition, studies have reported lower serum PK concentrations in patients with severe Alzheimer disease (21, 22) and more severe executive decline associated with those prescribed vitamin K agonists (23–25). Furthermore, increased vitamin K intake was associated with less frequent and severe subjective memory complaints in older adults (26). Despite recognition of the importance of vitamin K for various health parameters, including cognition, the impact of intestinally produced MK on cognition has not been fully elucidated. The objective of this study was to investigate associations between bacterially synthesized MK and cognition, determine if there were cognitive function-associated differences in the numbers and types of gut microbiota-encoded vitamin K biosynthesis genes and MK production, and assess whether altered gut microbiota composition was associated with these changes. We applied shotgun sequencing and quantification of MK concentrations to the microbial metagenome of 74 fecal samples from a well-characterized elderly Irish cohort to address this question.

## Methods

### Study participants

The study population was subsampled from the ELDERMET cohort, which was established in 2007 to determine the gut microbiota composition of 500 elderly individuals and associations with health (27, 28) (**Supplemental Figure 1**). Individuals were recruited as described previously (29), and ethical approval was obtained from the Cork Clinical Research Ethics Committee with written consent obtained for all subjects. ELDERMET subjects ranged in age from 64–93 y and were categorized based on where they resided as long stay (residing in long-term care facilities; *n* = 17), community (community dwelling; *n* = 41), rehab (in short-term rehabilitation care for <6 wk; *n* = 7), or day hospital (attending outpatient day hospital; *n* = 9). Exclusion criteria included alcohol abuse, participation in a drug intervention trial, advanced organic disease (e.g., cancer, heart disease, arthritis, gastric ulcers, and emphysema), and use of dietary supplements. Subjects prescribed medication known to influence the gut microbiota and vitamin K status, including antibiotics and anticoagulants, were also excluded.

### Data collection

Anthropometric measurements and medical and clinical history were collected. Cognitive ability was assessed using the Mini-Mental State Examination (MMSE) (30), which involves testing several aspects of cognitive function, including writing, short-term memory, orientation, and time. Subjects receive a score out of 30, with a higher score indicative of better cognitive function. All MMSE scores of 0 were replaced by the lowest nonzero MMSE score in the data set. An MMSE cutoff threshold of ≤24 was used to define mild to severe cognitive impairment (31). Recent nutritional PK intake was assessed by serum PK concentrations. Medical records were examined for information on diabetes and cardiovascular disease (risk factors for cognitive decline), and medication to treat these diseases and antipsychotics/antidepressants were treated as confounders as these could potentially influence the MMSE.

### DNA sequencing and data processing

Metagenomic sequencing of total fecal DNA was performed using the Illumina HiSeq 2000 instrument by generating 100-bp paired-end read libraries following the manufacturer's instructions. This resulted in an average of 21,955,822 read pairs per sample. Paired-end reads were quality filtered using Best Match Tagger [BMTagger (32)] to remove reads that matched the human genome. EstimateLibraryComplexity of the Picard suite of tools (http://picard.sourceforge.net) was used to remove duplicate reads. Low-quality reads were trimmed using trimBWAstyle.pl; any reads with length less than 60 bp were removed from downstream analysis. Reads were de novo assembled using Velvet v1.2.10 (33) with a k-mer size of 51 and the default parameters. Coding sequences were predicted, from the contigs, using the MetaGeneMark algorithm, which predicts genes using models that are based on dependencies, formed in evolution, between codon frequencies and genome nucleotide composition. A linear function relates nucleotide frequencies in the 3 codon positions to the global nucleotide frequencies, and linear functions also relate amino acid residue frequencies to genome guanine−cytosine content. The method is optimized with a specific formula to handle partial genes and can predict genes greater than 60 nucleotides (34). Predicted genes were annotated against the Kyoto Encyclopedia of Genes and Genomes (KEGG) (35) and Clusters of Orthologous Groups databases (36) using a Basic Local Alignment Search Tool cutoff e-value of 1 × 10^-5^ for the 74 samples.

### Quantification of serum phylloquinone, cytokine analysis, and fecal menaquinone

Blood and fecal samples were processed and stored following standard procedures as described elsewhere (27). Subjects were nonfasted and blood samples were taken by a trained nurse using a vacutainer. Blood samples were centrifuged, and after serum was extracted, samples were stored at −80°C. Serum phylloquinone was assessed by reverse-phase HPLC with fluorescence detection as described by Presse et al. (37). The inflammatory cytokines TNF-α, IL-6, and IL-8 (risk factors for cognitive decline) were measured using validated, commercial, multispot microplates (Meso Scale Diagnostics). Fecal menaquinone concentrations were also quantified by an Atmospheric-Pressure Chemical Ionization-LC/MS method described by Karl et al. (38).

### Creation of a menaquinone biosynthesis reference database

Publicly available MK biosynthesis genes, for both pathways, were downloaded from the KEGG database based on EC number. Representatives of the less common futalosine pathway were also downloaded from GenBank. To ensure a fully comprehensive data set, genes predicted using MetaGeneMark from the 74 ELDERMET assemblies were mined for MK biosynthesis genes, based on annotation, and pooled with the publicly available genes into an MK biosynthesis gene reference database. All gene sequences were clustered using UCLUST, of the USEARCH suite of tools (39), at 95% identity over 90% of the length to remove redundancy. This nonredundant gene data set served as a reference to which to align reads from the elderly individuals.

### Measurements of the abundance of MK biosynthesis genes in the ELDERMET data set

Quality trimmed reads from each of the 74 samples were aligned to the nonredundant MK reference database (created above) using the Bowtie2 alignment tool (40). Results were summarized using the Samtools’ idxstats method (41) to extract abundances from the alignments for each sample, and counts were normalized by library size to form the MK abundance matrix, which was subsequently analyzed in the R statistical package (v 3.1.2).

### Formation of vitamin K gene abundance clusters

Hierarchical clustering with Spearman distance and Ward's linkage was carried out on the MK gene abundance matrix in the R statistical package. The optimal number of clusters was determined based on the Calinsky-Harabasz index (42). A principal coordinates analysis (PCoA) (43) plot was inferred based on Spearman distances from the abundance matrix based on the number of each MK reference gene (as described above) in each sample. This method compares the rank of the gene abundance to generate a dissimilarity metric that can be used to ordinate samples in a multidimensional space where the axes (or eigenvectors) are ordered based on the amount of variance they explain. The result of this is that samples that share a similar abundance profile across the same reference genes will tend to be clustered more closely in the ordination plot.

Similarly, nonnegative matrix factorization (NMF) was also performed on the MK gene abundance matrix using raw counts and normalized raw counts with either square-root or log_10_ transformation. A regression analysis tested which axis among the first 10 axes from the PCoA and NMF exerted significant effects over the MMSE. To verify the clustering observed in the PCoA, principal components analysis (PCA) and NMF were inferred from the log_10_ normalized concentrations of the 10 MK isoforms.

### Statistical analysis

Statistical analysis was carried out using the R software (v 3.1.2). Statistically significant differences between groups were investigated, for nonparametric variables, using the Kruskal-Wallis test followed by a post hoc Dunn's test with Benjamini-Hochberg correction for multiple comparisons; these variables included MMSE scores, age, functional performance (Barthel score), multimorbidity, polypharmacy, serum PK concentrations, TNF-α, IL-6, IL-8, Shannon diversity, BMI, MK isoforms, steps of the MK pathway, and the first axis of PCoA (from MK gene abundance). Significant differences between the categorical variables (i.e., sex, strata, presence of hypertension, diabetes, and antidepression/antipsychotic medication) were evaluated using the χ^2^ test followed by Benjamini-Hochberg correction for multiple comparisons.

A multivariable-adjusted Poisson regression test was performed to examine the association between cognitive ability and vitamin K abundance clusters after adjusting for the following confounding factors: age; Barthel score; presence of hypertension and diabetes; number of cardiovascular and diabetes medications; polypharmacy; multimorbidity; concentrations of serum PK, TNF-α, IL-6, and IL-8; Shannon diversity; BMI; antipsychotic medications; sex; and strata. Individual multivariable-adjusted Poisson regression tests examined the association between each MK isoform and cognition and each step of the pathway and cognition after adjusting for clusters of all confounding factors. MMSE scores in each case were fed to the Poisson regression tests as the number of MMSE errors (i.e., 30-X, where X is the MMSE score of the patient and 30 is the maximum MMSE score). This generated a Poisson distribution of decreasing cognitive ability. Single unadjusted Poisson regression tests were used to determine the association between individual variables and MMSE score. Statistical differences between the vitamin K abundance clusters were determined using permutational multivariate ANOVA with the distance matrix using the Adonis function of the Vegan package in R. A *P* value of <0.05 was considered statistically significant. PCA was applied to identify confounding factors regulating the different cognitive ability levels of elderly individuals. A heatmap was generated by clustering samples based on the first 3 axes from the PCA of the confounding factors with Ward's minimum variance method. The association between MMSE and the vitamin K abundance clusters was further examined in a Poisson regression model in which all adjusted confounding factors were replaced by the clusters from the PCA of the confounding factors.

Redundancy analysis (RDA) was used to explore the association between MK isoforms and MMSE score adjusted for the effects of confounding factors. Statistical difference of the RDA model was calculated using an ANOVA. Correlation between MK isoforms was computed based on the Spearman rank correlation coefficient and was visualized as a network with qgraph R package (44).

### Calculating microbiota Shannon diversity of samples in the 4 clusters

The intrasample diversity (α diversity) was calculated for each sample in each of the 4 vitamin K abundance clusters using the paired ELDERMET 16S data set and the Vegan package within R. Data were visualized using boxplots.

### Taxonomic composition and differentially abundant taxa across the 4 clusters

The microbiota composition of each of the 74 fecal samples was derived using MetaPhlAn2 (45). Differential abundance of taxa between the 4 vitamin K abundance clusters was tested using the Wilcoxon rank-sum test with *P* values adjusted using the Benjamini-Hochberg correction.

## Results

### Descriptive statistics

Selected characteristics of the study participants are shown in **Table 1**. The study group comprised 51 females and 23 males. The mean MMSE score was 24, with 32% of patients having an MMSE score of ≤24, thus being classed as mild to severely cognitively impaired (46). The average age was 78 years with an average Barthel score of 15.7. Few patients were receiving hypertension or diabetes medication (reflected by the low mean numbers of medications taken); patients had an average of 3 diagnoses and were taking on average 5 drugs in total. The elderly subjects had an average blood serum phylloquinone concentration of 0.9 nmol/L. Concentrations of the inflammatory markers TNF-α, IL-6, and IL-8 averaged 6.7, 14.9, and 19.3 IU, respectively. Across the data set, microbiota diversity, as measured by the Shannon index, had an average value of 3.6 while BMI averaged 26.9.

**TABLE 1:** Descriptive characteristics of the study participants^1^

Characteristic	Mean ± SD	SEM
MMSE score	24 ± 6.6	0.77
Age, y	78 ± 8.7	1.02
Barthel score	15.7 ± 6.3	0.74
No. of hypertension medications	0.4 ± 0.8	0.09
No. of diabetes medications	0.1 ± 0.4	0.04
No. of diagnoses	3.2 ± 2.3	0.27
No. of drugs	5.0 ± 3.0	0.36
PK blood serum concentrations, nmol/L	0.9 ± 1.1	0.13
TNF-α, IU	6.7 ± 6.9	0.82
IL-6, IU	14.9 ± 33.2	3.95
IL-8, IU	19.3 ± 18.8	2.24
Diversity: Shannon index	3.6 ± 0.5	0.06
BMI, kg/m^2^	26.9 ± 6.3	0.73

1 Values are means **± **SD and SEM for *n* = 74 elderly samples; sex, *n*, male/female = 23/51. MMSE, Mini-Mental State Examination; PK, phylloquinone.

### Construction of a microbial vitamin K biosynthesis gene catalog

Menaquinone genes were downloaded from KEGG (classical MK and futalosine pathway) and GenBank [for the futalosine pathway (35)] for all steps of the MK biosynthesis pathway (**Supplemental Figure 2**). These genes were pooled with MK biosynthesis genes predicted, using MetaGeneMark, from the 74 ELDERMET assemblies to compile a nonredundant data set of 8688 MK biosynthesis genes. This served as a reference to align metagenomic sequence reads from the 74 ELDERMET subjects against, to determine the profile of the abundance of MK-producing genes in each individual (**Supplemental Table 1**). The number of reads from the 74 subjects that aligned to the MK reference database ranged from 21,454 to 64,067 per subject with an average number of 37,530 reads aligned per subject (**Supplemental Table 2**).

### Clustering analysis revealed discrete vitamin K biosynthesis gene abundance profile groups

**FIGURE 1:**
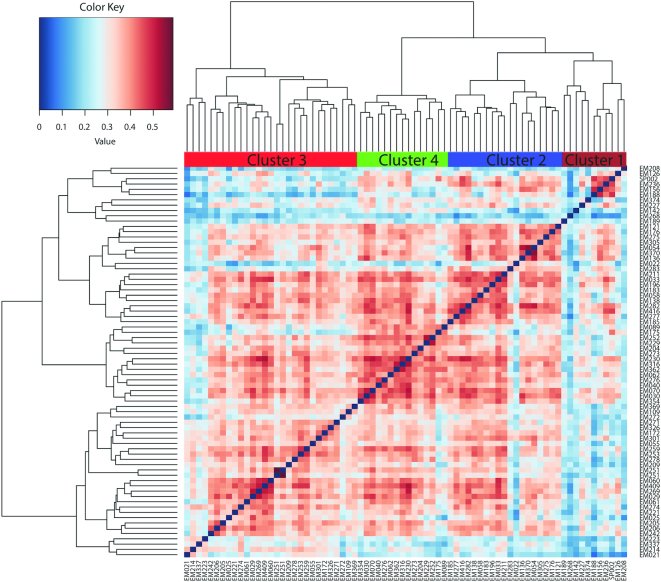
Hierarchical Ward-linkage clustering based on the Spearman correlation coefficients of the abundance of vitamin K genes for the 74 samples. Clusters are indicated by a color bar along the top of the heatmap and sample names along the rows and columns.

**FIGURE 2:**
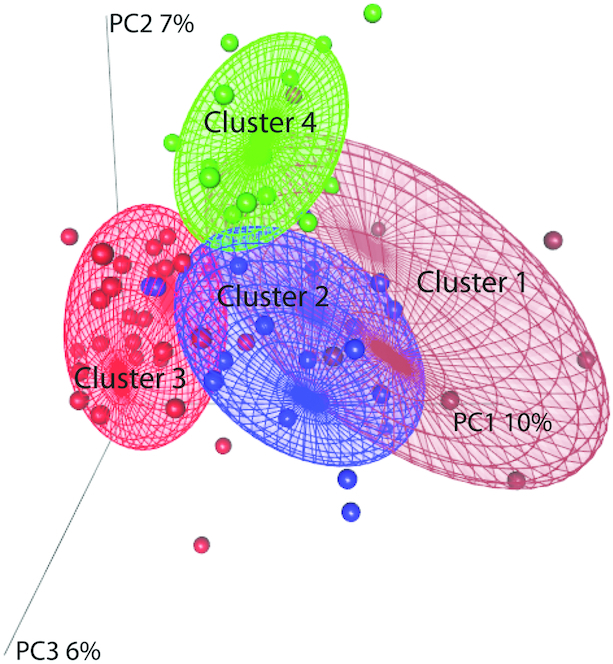
Principal coordinates analysis based on reads aligned to the vitamin K abundance matrix (VKAM), which shows the multivariate spread and variation of the VKAM across the subjects with significant differences observed across the 4 clusters (*P* ≤ 0.001, Adonis test). The first principal component (PC1) explains over 10% of the data set variation while the second PC explains 7%. Cluster 1 contained 11 subjects (15% of the samples), cluster 2 comprised 19 subjects (26%), cluster 3 had 29 subjects (39%), and cluster 4 contained 15 subjects (20%).

**FIGURE 3:**
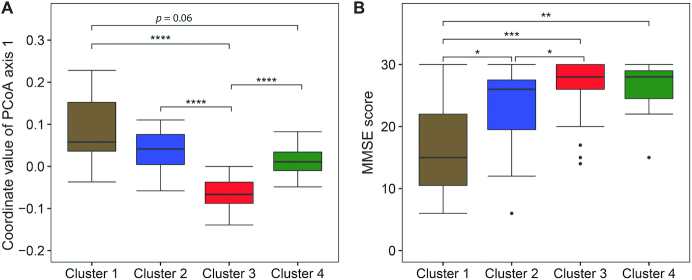
Distribution of samples’ coordinates along principal coordinates analysis (PCoA) axis 1 based on the menaquinone biosynthesis genes and Mini-Mental State Examination (MMSE) score differences across the 4 vitamin K abundance clusters. (A) Boxplots represent the distribution of samples’ coordinates, within each cluster, along the PCoA axis 1. Cluster 1 comprised 11 subjects, cluster 2 had 19 subjects, cluster 3 contained 29 subjects, and cluster 4 had 15 subjects. (B) Boxplots of the distribution of MMSE scores for the 74 samples, organized by vitamin K clusters. Cluster 1 comprised 11 subjects, cluster 2 had 19 subjects, cluster 3 contained 29 subjects, and cluster 4 had 15 subjects. Boxplots represent a 5-point summary of the data in the following order (from bottom to top): minimum, first quartile, median, third quartile, and maximum. *P* values were calculated by Kruskal-Wallis tests with post hoc Dunn test using Benjamini-Hochberg correction. *P < 0.05. **P < 0.01. ***P < 0.001. ^****^P < 0.0001.

To identify subjects/fecal samples with similar concentrations of predicted MK production capacity, the vitamin K biosynthesis gene abundance matrix (VKAM) was subjected to hierarchical clustering. This clustering was performed based on Ward's linkage method, which clusters samples based on similarities in the presence and abundance of their MK reference genes; in other words, within-cluster samples share a more similar abundance profile. As shown in **Figure 1**, this analysis identified 4 clusters hereafter referred to as clusters 1–4. Four clusters were considered the optimal to provide a high resolution while retaining a good sample size per group using the Calinsky-Harabasz index (42) (**Supplemental Figure 3**). PCA revealed the multivariate spread and variation of the VKAM across the subjects (**Figure 2**) with significant differences observed across the 4 clusters (*P* ≤ 0.001, Adonis test). The first principal component (PC1) explains over 10% of the data set variation while the second PC explains 7%. Cluster 1 contained 11 subjects (15% of the samples), cluster 2 comprised 19 subjects (26%), cluster 3 had 29 subjects (39%), and cluster 4 contained 15 subjects (20%). NMF inferred from log_10_ transformation of the VKAM also supported the 4 clusters (**Supplemental Figure 4**). An investigation into the spread of the coordinates of the samples along PCoA axis 1 (accounting for the largest variance) revealed that cluster 3 samples were significantly different from the 3 other clusters based on Kruskal-Wallis tests with post hoc Dunn test using Benjamini-Hochberg correction (**Figure 3**A). PCA and NMF inferences were also constructed from the concentrations of the 10 MK isoforms (**Supplemental Table 3** and **Supplemental Table 4**) and supported clustering of the samples into 4 clusters (**Supplemental Figure 5**).

### Justification of the use of PCoA over NMF

A regression analysis to test which axis, among the first 10 axes, from the PCoA and NMF (based on the VKAM using raw counts and normalized raw counts with either square-root or log_10_ transformation) exerted significant effects over the MMSE revealed for the PCoA that axes 1, 5, and 6 were significantly associated with the MMSE (**Supplemental Tables 5** and **6**). For the NMF constructed using raw counts, axis 7 was significantly associated, while for square-root and log_10_ transformations of the raw counts, axes 2 and 1, respectively, were significantly associated with the MMSE.

### Distribution of descriptive variables across the clusters

Descriptive statistics of clinical metadata averages by cluster are shown in **Table 2** and the individual values in **Supplemental Table 7**. The values for BMI, serum concentrations of the inflammatory cytokine IL-8, number of hypertension medications, and total drug intake were similar across the 4 clusters (*P* > 0.05; Table 2). In contrast, MMSE scores differed significantly across the clusters (*P* = 0.001, Table 2) with cluster 3 characterized by the highest average MMSE score and cluster 1 the lowest average MMSE score (*P* < 0.001; Supplemental Table 7; Table 2). Age also differed significantly across the 4 clusters (*P* = 0.019). Cluster 1 was characterized by the highest average age and differed significantly from clusters 3 and 4, with cluster 4 having the lowest average age (*P* = 0.015 and 0.010, respectively; Table 2; Supplemental Table 7). Serum PK concentrations were significantly different across the clusters (*P* = 0.04), but pairwise cluster comparison failed to reach significance (Table 2). Subjects in cluster 1 had higher TNF-α concentrations and number of diagnoses (*P* = 0.018; *P* = 0.001; respectively; Table 2; Supplemental Table 7) than those in cluster 3. There was also a significant difference in Shannon diversity and Barthel score across the VKAM clusters (*P* *=* 0.001 and *P* < 0.001; Table 2), with samples in cluster 1 having a lower average score than cluster 3. Subjects in cluster 1 differed significantly in their residential location from subjects in cluster 3 (*P* = 0.020) and cluster 4 (*P* = 0.006; **Table 3**). Cluster 1 was mainly characterized by subjects residing in long-stay accommodation (64% of samples; Supplemental Table 7) with some community and rehab residents (18% in both cases). Clusters 3 and 4 mainly comprised community residents (63% and 80%, respectively; Supplemental Table 7), with fewer long-stay (13% and 0%), day-hospital (13% and 13%), and rehab (10% and 6.7%) residents. Almost half of cluster 2 individuals were community dwellers (47%) with 31% residing in long-stay accommodation.

**TABLE 2:** Characteristics of the study's participants organized by vitamin K abundance clusters^1^

	Mean ± SD					Cluster vs. cluster *P*^2^					
Characteristic	Cluster 1 (*n* = 11)	Cluster 2 (*n* = 29)	Cluster 3 (*n* = 11)	Cluster 4 (*n* = 15)	*P* ^3^	1 vs. 2	1 vs. 3	1 vs. 4	2 vs. 3	2 vs. 4	3 vs. 4
MMSE score	15 ± 9.7	23 ± 7.5	27 ± 4.6	26 ± 4	0.001^3^	0.035^3^	<0.001^3^	0.001^2^	0.032^2^	0.123	0.272
Age, y	85 ± 7.4	80 ± 9.6	76 ± 8.3	75 ± 6.8	0.019^3^	0.121	0.015^2^	0.010^2^	0.100	0.118	0.400
Barthel score	8.3 ± 6.5	14.7 ± 7.1	17.4 ± 4.9	18.5 ± 3.6	<0.001^3^	0.025 ^2^	<0.001^2^	0.042^2^	<0.001^2^	0.039^2^	0.323
No. of hypertension medications	0.18 ± 0.4	0.53 ± 0.9	0.45 ± 0.9	0.27 ± 0.6	0.759	1.000	0.508	0.433	0.471	0.594	0.480
No. of diabetes medications	0 ± 0	0.21 ± 0.5	0.07 ± 0.3	0 ± 0	<0.001^3^	0.105	0.396	0.500	0.071^2^	0.140	0.469
No. of diagnoses	5.9 ± 2.9	3.7 ± 2	2.2 ± 1.8	2.8 ± 2	0.001^3^	0.090	0.001^2^	0.012^2^	0.014^2^	0.112	0.188
No. of drugs	6.3 ± 3.2	4.7 ± 2.9	4.8 ± 3.6	4.8 ± 1.9	0.339	0.199	0.220	0.413	0.221	0.485	0.480
Serum PK, nmol/L	0.82 ± 08	0.86 ± 0.8	1.18 ± 1.5	0.73 ± 0.5	0.04^3^	0.582	0.347	0.489	0.720	0.706	0.468
TNF-α, IU	7.89 ± 3.7	9.97 ± 12.5	4.79 ± 1.9	5.64 ± 2.8	0.033^3^	0.177	0.018^2^	0.083	0.091	0.240	0.191
IL-6, IU	13.13 ± 12.7	28.08 ± 51.1	5.96 ± 3.5	17.67 ± 42.8	0.025^3^	0.351	0.101	0.221	0.013^2^	0.087	0.252
IL-8, IU	25.56 ± 17.5	18.59 ± 8.4	20.38 ± 26.4	14.42 ± 7.8	0.198	0.2392	0.2003	0.1318	0.2237	0.1486	0.2878
Diversity: Shannon index	3.04 ± 0.4	3.53 ± 0.3	3.79 ± 0.5	3.55 ± 0.6	0.001^3^	0.017^2^	<0.001^2^	0.018^2^	0.051^2^	0.499	0.058
BMI, kg/m^2^	23.7 ± 5.8	27 ± 4.5	26.5 ± 5.8	29.8 ± 8.8	0.123	0.047	0.083	0.290	0.091	0.439	0.316

1 Values are means ± SD. Individual values can be found in Supplemental Table 7. MMSE, Mini-Mental State Examination; PK, phylloquinone.

2 *P* < 0.05, Dunn post hoc test for pairwise comparisons.

3 *P *< 0.05, Kruskal-Wallis test.

**TABLE 3:** Categorical characteristics of the study's participants organized by vitamin K abundance clusters^1^

	Number					Cluster vs. cluster *P*^2^					
Characteristic	Cluster 1 (*n* = 11)	Cluster 2 (*n* = 19)	Cluster 3 (*n* = 29)	Cluster 4 (*n* = 15)	*P* ^3^	1 vs. 2	1 vs. 3	1 vs. 4	2 vs. 3	2 vs. 4	3 vs. 4
Sex, *n*					0.963	1	1	0.920	1	1	1
Male/female	4/7	6/13	9/20	4/11							
Strata, *n*					0.013^3^	0.155	0.020^2^	0.006^2^	0.463	0.155	0.463
Long stay	7	6	4	0							
Rehabilitation	2	1	3	1							
Day hospital	0	3	4	2							
Community	2	9	18	12							
Hypertension, *n*					0.359	0.973	1	1	0.973	0.973	1
Presence/absence	3/8	9/10	7/22	4/11							
Diabetes, *n*					0.036^3^	0.562	1	1	0.525	0.525	1
Presence/absence	0/11	4/15	1/28	0/15							
Antipsychotic medications, *n*					0.094	0.275	0.275	0.423	1	1	1
Presence/absence	6/4	4/15	6/23	4/11							

1 Values are numbers for each variable detailing the categorical characteristics of *n* = 74 elderly samples. Individual values can be found in Supplemental Table 7.

2 *P* < 0.05 Benjamini-Hochberg for multiple corrections.

3 *P* < 0.05 χ^2^ test.

### MMSE associations with individual variables

Individual clinical variables were assessed for their association with cognitive function using single unadjusted Poisson regression tests (**Supplemental Table 8**). Age, residing in long-stay or rehab facilities, higher concentrations of TNF-α, IL-6, and IL-8, and a higher number of diagnoses, drugs (in general), and antidepressants/antipsychotics were all associated with increases in MMSE errors while a presence of hypertension and a higher Shannon diversity value, BMI, and Barthel score were found to be positively associated with a decrease in MMSE errors. Interestingly, serum PK was significantly associated with MMSE (Supplemental Table 8), but when confounding factors were added to the model (**Table 4**), this relation was no longer significant. Residing in a day hospital and being male or diabetic were not found to be significantly associated with cognition (*P* > 0.05; Supplemental Table 8).

**TABLE 4:** Association of each variable in the Poisson statistical model with MMSE^1^

Variable	e^RC^	2.5%, 97.5% CI	*P* value
Age, y	1	0.98, 1.02	0.98
Cluster 2 (*n* = 19)	0.58	0.38, 0.89	0.013^2^
Cluster 3 (*n* = 29)	0.54	0.33, 0.89	0.017^2^
Cluster 4 (*n* = 15)	0.83	0.51, 1.37	0.47
Strata (day hospital) (*n* = 9)	1.32	0.59, 2.77	0.48
Strata (long stay) (*n* = 17)	3.38	1.51, 7.55	0.003^2^
Strata (rehab) (*n* = 7)	3.15	1.59, 6.26	<0.001^2^
Gender (male) (*n* = 23)	0.89	0.62, 1.27	0.52
Barthel score	0.98	0.93, 1.03	0.49
No. of diagnoses	0.91	0.83, 1	0.049^2^
No. of drugs	0.98	0.91, 1.05	0.48
Presence of hypertension (*n* = 23)	0.87	0.54, 1.38	0.58
Hypertension, 1 medication (*n* = 8)	0.83	0.47, 1.47	0.52
Hypertension, 2 medications, (*n* = 6)	1.55	0.69, 3.42	0.28
Hypertension, 3 medications, (*n* = 3)	0.55	0.08, 2.07	0.45
Presence of diabetes (*n* = 5)	4.17	0.58, 32.46	0.16
Diabetes, 1 medication (*n* = 2)	0.26	0.03, 2.02	0.21
Diabetes, 2 medications (*n* = 2)	0.31	0.03, 3.26	0.33
Serum PK, nmol/L	0.93	0.65, 1.25	0.64
TNF-α, IU	0.99	0.95, 1.04	0.73
IL-6, IU	1.01	1, 1.01	0.055
IL-8, IU	1	0.99, 1	0.61
Diversity: Shannon index	0.74	0.5, 1.11	0.14
BMI, kg/m^2^	0.99	0.95, 1.02	0.4
Antidepressants/antipsychotics (*n* = 20)	1.52	0.97, 2.38	0.066

1 Association between the vitamin K clusters and MMSE after adjusting for age; Barthel score; presence of hypertension and diabetes; number of cardiovascular and diabetes medications; polypharmacy; multimorbidity; concentrations of serum PK, TNF-α, IL-6, and IL-8; Shannon diversity; BMI; antipsychotic medications; gender; and strata. e^RC^, exponentiated regression coefficient; MMSE, Mini-Mental State Examination; PK, phylloquinone.

2 *P* < 0.05, Poisson regression model.

### MMSE association with vitamin K abundance clusters and MK biosynthesis genes

Multivariable-adjusted Poisson regression tests were performed to examine the association between cognitive ability and the VKAM clusters after adjusting for the following confounding factors: age; Barthel score; presence of hypertension and diabetes; number of cardiovascular and diabetes medications; polypharmacy; multimorbidity; concentrations of serum PK, TNF-α, IL-6, and IL-8; Shannon diversity; BMI; antipsychotic medications; sex; and strata (Table 4). A statistically significant association was observed between cluster 2, cluster 3, and the number of diagnoses and a decrease in the number of MMSE errors (Table 4; *P* = 0.013 and 0.017, respectively). The spread of MMSE within each of the 4 VKAM clusters was displayed as boxplots with the median and interquartile ranges indicated (Figure 3B). Residing in long-stay (*P* = 0.003) or rehab care (*P* < 0.001) facilities was inversely associated with the number of MMSE errors. The association between MMSE and MK biosynthesis genes was further examined as described in the **Supplemental Text**.

### Hierarchical clustering revealed a simplified structure of all confounding factors and the consistent association of MMSE and vitamin K abundance clusters

To obtain a simplified structure of all confounding factors, the profiles of the confounding factors were subjected to PCA and hierarchical clustering. The first component of the PCA separates samples by frailty, comorbidity, and serum PK, whereas the second and third components correlate with hypertension, diabetes, BMI, and sex (**Supplemental Figure 6**A). Hierarchical clustering performed with 51.5% of the data variance from the first 3 components revealed 3 separate clusters that were able to distinguish long-stay/rehab and community/day hospital (Supplemental Figure 6B). The association between MMSE and vitamin K abundance clusters was further examined in a Poisson regression model in which all adjusted confounding factors were replaced by the clusters from the PCA of the confounding factors. Vitamin K abundance cluster 3 remained significant (*P* < 0.001) while a statistically significant difference was also observed in cluster 4 (*P* < 0.001; **Supplemental Table 9**).

### Association of MK isoforms with clusters and MMSE

The distribution of MK isoforms, with the exception of MK4, MK6, and MK7, differed significantly across the clusters (**Table 5**). MKs 9 and 10 were in the highest abundance in cluster 1 (Supplemental Table 3), with the former differing from clusters 3 and 4 (*P* = 0.010 and *P* < 0.001) and MK10 from cluster 4 (*P* = 0.001; Table 5). MK8 was more highly abundant in cluster 2 than in clusters 3 and 4 (*P* = 0.001 and *P* < 0.001, respectively; Supplemental Table 3; Table 5). MKs 5, 12, and 13 were higher in cluster 3 (Supplemental Table 3) with all isoforms differing from cluster 1 (*P* = 0.003, *P* = 0.002, *P* < 0.001). MK concentration was also assessed for an association with MMSE after adjusting for clusters of confounding factors, and MKs 4, 8, 9, and 10 were found to have a negative association while MKs 6, 12, and 13 were found to have a positive association, indicating these isoforms are the most important for cognition (**Table 6**).

**TABLE 5:** Results of Kruskal-Wallis tests to assess differences across the clusters for each of the MK chains^1^

			Cluster vs. cluster *P*^2^					
Characteristic	*P* ^3^	BH-adjusted *P* value^4^	1 vs. 2	1 vs. 3	1 vs. 4	2 vs. 3	2 vs. 4	3 vs. 4
MK4	0.610	0.610	0.466	0.639	0.438	0.417	0.371	0.465
MK5	<0.001^3^	<0.001^4^	0.016^2^	0.003^2^	0.293	0.324	0.002^4^	<0.001^2^
MK6	0.074	0.102	0.039	0.059	0.157	0.487	0.176	0.209
MK7	0.289	0.317	0.440	0.271	0.298	0.172	0.319	0.391
MK8	<0.001^3^	<0.001^4^	0.389	0.011^2^	<0.001^2^	0.001^2^	<0.001^2^	0.009^2^
MK9	<0.001^3^	<0.001^4^	0.464	0.010^2^	<0.001^2^	0.001^2^	<0.001^2^	0.017^2^
MK10	0.001^3^	0.001^4^	0.191	0.068	0.001^2^	0.215	0.002^2^	0.007^2^
MK11	<0.001^3^	0.001^4^	0.328	0.271	0.012^2^	0.349	0.001^2^	<0.001^2^
MK12	<0.001^3^	0.001^4^	0.110	0.002^2^	0.429	0.035^2^	0.091	0.001^2^
MK13	<0.001^3^	0.001^4^	0.062	<0.001^2^	0.199	0.015^2^	0.207	0.003^2^
PK	0.182	0.222	0.174	0.094	0.260	0.328	0.327	0.223

1 Results of assessing the independence of MK chain concentration across the clusters. Cluster 1, *n* = 11; cluster 2, *n* = 19; cluster 3, *n* = 29; cluster 4, *n* = 15. Individual MK and PK values are listed in Supplemental Tables 3 and 7, respectively. BH, Benjamini-Hochberg; MK, menaquinone; PK, phylloquinone.

2 *P* < 0.05, Dunn post hoc test for pairwise comparisons, adjusted by the BH method.

3 *P* < 0.05, Kruskal-Wallis test.

4 *P* < 0.05, Kruskal-Wallis test adjusted using BH correction.

**TABLE 6:** Poisson regression model examining the association of MK chains and MMSE^1^

MK chain	e^RC^	2.5, 97.5% CI	*P* value
MK4	1.1	1.04, 1.17	0.001^2^
MK5	1.31	0.61, 2.73	0.48
MK6	0.85	0.75, 0.94	0.003^2^
MK7	1.01	0.96, 1.06	0.72
MK8	1.01	1, 1.02	<0.001^2^
MK9	1.21	1.14, 1.28	<0.001^2^
MK10	1.06	1.03, 1.08	<0.001^2^
MK11	1	0.97, 1.03	0.92
MK12	0.98	0.96, 0.99	0.009^2^
MK13	0.99	0.98, 1	0.014^2^

1 Association between each MK isoform and Mini-Mental State Examination after adjusting for the clusters inferred from all confounding factors. Only MK variables are shown. e^RC^, exponentiated regression coefficient; MK, menaquinone.

2 *P* < 0.05; Poisson regression model.

RDA of the relation between MK isoforms and MMSE score indicated that MK8 and MK13 were the major factors driving the variation in MMSE score. MMSE score was positively correlated with MK12 and MK13 and negatively correlated with MK4, MK7, MK8, MK9, and MK10 (**Supplemental Figure 7A**). Additionally, the correlation analysis among MK isoforms revealed the strong correlation between MK8, MK9, and MK10, as well as between MK5, MK11, MK12, and MK13 (Supplemental Figure 7B and **Supplemental Table 10**).

### Association of each step of the vitamin K pathway with clusters and MMSE

Across the clusters, the microbiome gene counts for encoding *MenD, MenH*, and *MenC* were significantly different, with *MenD* being highest in cluster 3 and *MenH* and *MenC* highest in cluster 2, but were no longer significant upon adjusting for multiple testing (**Table 7** and **Supplemental Table 11**). A Poisson regression model, which adjusted for clusters of confounding factors, showed that 2 steps/enzymes were significantly associated with MMSE scores, namely, *MenF* and *MenI* (**Table 8**).

**TABLE 7:** Results of Kruskal-Wallis examining the spread of the pathway steps across the clusters^1^

Steps	*P* value	BH-adjusted *P* value
Futalosine	0.146	0.245
MenF	0.065	0.163
MenD	0.041^2^	0.138
MenH	0.007^2^	0.075
MenC	0.023^2^	0.119
MenE	0.112	0.226
MenB	0.252	0.361
MenI	0.319	0.399
MenA	0.783	0.871
MenG	0.872	0.873

1 Examining the spread of the menaquinone biosynthesis pathway genes across the clusters. Cluster 1, *n* = 11; cluster 2, *n* = 19; cluster, 3 *n* = 29; cluster 4, *n* = 15. Kruskal-Wallis test adjusted using BH correction. Individual counts for each of the steps are listed in Supplemental Table 11. BH, Benjamini-Hochberg.

2 *P* < 0.05, Kruskal-Wallis test.

**TABLE 8:** Association of each step of the vitamin K pathway and MMSE^1^

Step	e^RC^	2.5, 97.5% CI	*P* value
Futalosine	1	0.9998, 1.0001	0.63
MenF	0.9999	0.9998, 1	0.003^2^
MenD	1	0.9999, 1	0.26
MenH	0.9997	0.9993, 1.0001	0.12
MenC	0.9998	0.9995, 1.0001	0.21
MenE	1	1, 1	0.7
MenB	0.9999	0.9998, 1	0.076
MenI	1.0336	1.0151, 1.0512	<0.001^2^
MenA	1	0.9999, 1.0001	0.96
MenG	1.0001	1, 1.0002	0.16

1 Associations between steps of the vitamin K pathway and MMSE errors after adjusting for clusters of confounding factors for *n* = 74 samples. e^RC^, exponentiated regression coefficient; MMSE, Mini-Mental State Examination.

2 *P* < 0.05, Poisson regression model.

### Vitamin gene clusters have different gene counts and microbiota diversity

A reduction in microbiota diversity or intestinal microbiota gene count has been associated with conditions including obesity (47), rheumatoid arthritis (48), inflammatory bowel disease (49), and increased frailty in elderly people (27). The microbiota diversity of samples in each of the VKAM clusters was assessed using the Shannon diversity index, which gives a quantitative measure of how many different species are in a sample. Data were visualized by boxplots, with the median and interquartile ranges indicated (**Supplemental Figure 8**). The microbiota diversity of the samples differed significantly between the VKAM clusters (Table 2, *P* = 0.001), with samples in cluster 3 showing the highest microbiota diversity while samples in cluster 1 had the lowest (*P* < 0.001; Supplemental Table 7). The normalized gene count was also lower in long-stay individuals (27) and was associated with decreased microbiota diversity. It is therefore not surprising that cluster 1 had the lowest gene counts across the samples while cluster 3 had the highest (**Supplemental Figure 9**). We have adjusted for diversity in the models to show that it does not fully explain the association between MK and MMSE.

### Taxonomic composition and differentially abundant taxa across the 4 clusters

Differential abundance of taxa between the 4 vitamin K abundance clusters was tested using the Wilcoxon rank-sum test (**Supplemental Table 12**). *Barnesiella* and *Odoribacter* were found to be present in lower abundance in cluster 1 than in cluster 2 (*P* < 0.05). These 2 genera, along with *Paraprevotella (P* < 0.05), *Coprococcus* (*P* < 0.05), and *Prevotella* (*P* < 0.05), were present in lower abundance in cluster 1 than in cluster 3, with only *Eggerthella* present at significantly higher concentrations in cluster 1 (*P* < 0.05). In addition, cluster 4 had a higher prevalence, along with *Barnesiella* (*P* < 0.01) and *Odoribacter* (*P* < 0.05), of *Haemophilus* (*P* < 0.01) and lower *Clostridium* (*P* < 0.01) compared with cluster 1. The abundance of *Escherichia* was higher in cluster 2 compared with clusters 3 and 4 (*P* < 0.001).

## Discussion

The primary objective of the ELDERMET study (http://eldermet.ucc.ie) was to investigate the interaction between the gut microbiota, diet, and health in a cohort of older Irish individuals. A subset of the ELDERMET cohort was selected for the current study to investigate associations between MK biosynthesis and cognitive function (determined by the MMSE). The primary finding of this study is that, irrespective of all measured potential confounders, variation in the MK biosynthesis concentration is associated with significant differences in MMSE scores (Table 2 and Table 4) driven by certain MK chain isoforms (Table 6) and steps of the MK pathway (Table 8). Positive associations between cognition and PK have been assessed based on FFQ information (50), and lower serum PK concentrations have been reported in patients with severe Alzheimer disease (21, 22). However, the potential interaction of the genes involved in MK biosynthesis, the encoded MKs, and cognition has never been examined.

Based on clustering of the VKAM by PCoA, we found 4 optimal clusters (Figure 2). To assess the robustness of the PCoA, NMF was also carried out on the VKAM along with normalized raw counts with square-root and log_10_ transformations. A linear regression analysis between the first 10 axes in each case and MMSE revealed PCoA exerted the greatest effects over MMSE, with axes 1, 5, and 6 significantly associated (Supplemental Table 6). PCA and NMF inferences from the concentrations of the 10 MK isoforms (Supplemental Table 4) also supported clustering of the samples into 4 clusters (Supplemental Figure 5).

Subjects harboring the cluster 1 MK biosynthesis genes were associated with poorer health (including the lowest MMSE score) in general and, in particular, when compared with cluster 3 (highest MMSE scores; Figure 3B and Table 2). Cluster 1 subjects had a higher TNF-α [Supplemental Table 7; linked to dementia in centenarians (51)], a lower microbial Shannon diversity [associated with rheumatoid arthritis (48) and increased frailty (28)], a lower Barthel score, and a higher proportion residing in long-term care (64% compared with 14%) and number of diagnoses compared with cluster 3 (Supplemental Table 7 and Table 2). An investigation of the coordinates of the samples along PCoA axis 1 revealed that cluster 3 samples were significantly different from the 3 other clusters (Figure 3A). Taken together, this evidence suggests that subjects harboring the cluster 1 MK biosynthesis genes are typified by a number of negative health parameters, which are reflective of the MK biosynthesis concentration and the MKs produced.

To our knowledge, we have shown, for the first time, that certain MK isoforms have a positive association with MMSE—namely, MKs 6, 12, and 13 (Table 6; Supplemental Figure 7)—indicating these isoforms are important for cognitive function, whereas MKs 4, 8, 9, and 10 have a negative association. While this work does not prove a causal effect of bacterial MK synthesis on cognition, it is interesting that certain MK isoforms were associated with specific clusters, with only MKs 4, 6, and 7 being similar across all clusters. Cluster 1 samples contained the highest abundance of MK9 and MK10 (Supplemental Table 3), and a clustering of samples enriched in these MKs was also observed by Karl et al. (52), who delineated samples into so-called menaquinotypes, based on the variability in fecal vitamin K content. Conversely, MKs 5, 12, and 13 were highest in cluster 3, with all isoforms differing from cluster 1 (Table 5). Samples enriched in these MKs formed the second menaquinotype, indicating vitamin K biosynthesis genes can be studied to identify menaquinotypes (52).

To elucidate further, we investigated which MK biosynthesis enzymes were driving the association with MMSE scores. The genes *MenF* and *MenI* of the classical MK pathway were significantly associated with MMSE scores (Table 8), but no steps were significantly different across the clusters after adjusting for multiple testing (Table 7).

Karl et al. (38, 52) showed that the variation in MK production by a cohort fed an intervention diet was attributed to differences in the fecal content of several bacterially synthesized MKs and not to the dietary forms of vitamin K (i.e., MK4 and PK). While mechanisms of MK absorption from the colon are currently unknown, these results suggest that fecal MK content can be altered by dietary change. Few genera were responsible for determining the variability in fecal MK6 and MK8–13. Our findings show a number of these genera are differentially abundant between the VKAM clusters (Supplemental Table 12). For example, *Prevotella* (one of the most significant taxa in defining menaquinotypes), which produces MKs 11, 12, and 13 (53); *Paraprevotella*, which produces MKs 10, 11, 12, and 13 (54); and *Barnesiella*, which produces MKs 11 and 12, are more abundant in cluster 3 than cluster 1 (Supplemental Table 12). It is therefore unsurprising that cluster 3 had the highest abundance of MKs 11, 12, and 13 (Supplemental Table 3). Cluster 2 was the only cluster to present with a higher abundance of *Escherichia* compared with clusters 3 and 4. This genus has been associated with MK 8 (12) and corroborates with a significantly higher abundance of MK8 in cluster 2 than clusters 3 and 4 (Table 5). Moreover, as shown previously, the strong correlations observed between specific MK isoforms (see correlation network, Supplemental Figure 7B) could be due to coproduction by the same bacterial taxa (e.g., *Prevotella* produces MKs 11, 12, and 13) (52, 53).

The question still remains: what proportion of gut bacterial products is available for absorption? It has been shown, looking at different sites along the human intestine, that the majority of MKs are present in the distal colon (55). Given their involvement in prokaryotic electron transport, most MKs are thought to be embedded in the bacterial cytoplasmic membrane. MK absorption has been postulated, although not well characterized, to occur via a bile acid–dependent pathway (56), with the most likely site of absorption being the terminal ileum where bile acids are present, as are some MK-producing bacteria, albeit at a lower concentration than in the colon (57). However, evidence exists to support the notion that at least some distal gut bacteria–produced MKs are absorbed; for example, the presence of MKs 10–13 in the human liver at high concentrations (11) suggests a bacterial origin for these forms at the very least (58). Animal models have shown that MK absorption from the terminal ileum and colon occurs by passive diffusion (59), with different routes of absorption applying to different isoforms (e.g., the portal route for long-chain MKs compared with the lymphatic route for shorter-chain MKs and PK) (60). While it remains to be fully elucidated, long-chain MKs have been shown to have a longer half-life than shorter-chain isoforms and PK, suggesting plasma kinetics may extend the bioavailability of long-chain MK for extrahepatic tissue uptake (56). While previous studies have reported higher serum PK concentrations were positively associated with verbal episodic memory (as measured by the cognitive test battery) in an aging population (37), evidence from the current study does not support a significant association between serum PK concentrations and cognition (Table 4). While the MMSE (which assesses writing, short-term memory, orientation, and time) is an excellent first-level screening tool, its usage as the sole instrument is an important limitation of the current study as it is not designed to determine subtle differences in cognitive function as accurately as other tests, and it has relatively low sensitivity for mild symptoms. Future work should include more comprehensive measures exploring specific aspects of cognitive functioning and investigating further potential covariates that are considered to have a statistical relation with gut microbiota composition in multivariate statistical analyses. Furthermore, the cross-sectional design of the current study is another limitation that does not allow inference of cause and effect; therefore, the findings reported herein must be interpreted with caution.

The variances reported herein infer potential differences in specific MK production and important physiological roles within the brain. Although the direction and possible causality of this association remain unknown, the observed associations highlight the desirability of a greater understanding of the relation between cognition and gut-derived MK.

## Supplementary Material

nqz220_Supplemental_Files

## Notes

This work was funded by the US Allen Foundation. The Allen Foundation had no role in the design, implementation, analysis, and interpretation of data associated with this study. EMOC's laboratory is also funded by the Department of Agriculture, Food and the Marine under the Food Institute Research Measure (17F521), the Health Research Board, and the Irish Heart Foundation. IBJ is supported by a Science Foundation Ireland grant (13/SIRG/2128). This work was supported, in part, by Science Foundation Ireland through a Center award to APC Microbiome Ireland (SFI/12/RC/2273). SLB's work is supported by the USDA Agricultural Research Service under Cooperative Agreement No. 58-1950-7-707. Any opinions, findings, conclusions, or recommendations expressed in this publication are those of the authors and do not necessarily reflect the views of the USDA.

Supplemental Figures 1–9, Supplemental Tables 1–12, and Supplemental Text are available from the “Supplementary data” link in the online posting of the article and from the same link in the online table of contents at https://academic.oup.com/ajcn/.

Abbreviations used: KEGG, Kyoto Encyclopedia of Genes and Genomes; MK, menaquinone; MMSE, Mini-Mental State Examination; NMF, nonnegative matrix factorization; PC, principal component; PCA, principal components analysis; PCoA, principal coordinates analysis; PK, phylloquinone; RDA, redundancy analysis; VKAM, vitamin K abundance matrix.
