# Supplementary material for: Exploratory analysis of covariation of microbiota-derived vitamin K and cognition in older adults
Source: Am J Clin Nutr. 2019 Sep 13;110(6):1404–15. doi: 10.1093/ajcn/nqz220 (PMC6885478; doi:10.1093/ajcn/nqz220)
Supplement: nqz220_Supplemental_Files [file nqz220_supplemental_files.zip › McCann_etal_Supplemental_Text.docx]

**Exploratory analysis of covariation of microbiota-derived vitamin K and cognition in older adults**

### Angela McCann, Ian B. Jeffery, Bouchra Ouliass, Guylaine Ferland, Xueyen Fu, Sarah L. Booth, Tam T. T. Tran, Paul W. O’Toole, Eibhlís M. O’ Connor

**Supplemental Text. MMSE association with MK biosynthesis genes**

**Method**

A reduced Poisson regression model examined the association between the microbiota derived vitamin K genes though the first axis of PCoA, from MK gene abundance, and MMSE while adjusting for residence strata and a single confounding factor in each instance. A reduced linear regression analysis assessed the association between the PCoA axis 1, based on the MK biosynthesis genes, and MMSE effects adjusting for individual confounding factors that drive gut microbiota variation.

**Result**

To validate the association between MMSE and MK biosynthesis genes, the four VKAM clusters were removed and replaced by PCoA axis 1 from MK gene abundances. A reduced regression model between MMSE and PCoA axis 1, wherein a single variable acted as a cofounder in each case, revealed this association was significant when adjusting for being male, presence of hypertension, diabetes and antidepressants/antipsychotics, serum PK, IL-6, IL-8, BMI, Barthel score and number of drugs. Significance was lost for the remaining co-founders (age, presence of hypertension, hypertension medications, presence of diabetes, diabetes medication, TNF-alpha, Shannon diversity and number of diagnoses; **Table 1**).

In addition a reduced linear regression analysis to examine the association between the PCoA axis 1, based on the MK biosynthesis genes, and MMSE effects adjusting for individual confounding factors that drive gut microbiota variation found that the association between MMSE and the MK genes was significant for all variables with the exception of when adjusted for age, frailty-related (Barthel score), strata and number of diagnoses (**Table 2**).

**Discussion**

The reduced regression model between PCoA axis 1 and MMSE revealed this association was still significant after adjusting for being male, antidepressants/antipyschotics, serum PK, IL-6, IL-8, BMI, Barthel score and number of drugs. The loss of significance for the remaining co-founders (age, presence of hypertension, hypertension medications, presence of diabetes, diabetes medication, TNF-alpha, shannon diversity and number of diagnoses; **Table 1**) may be due to interaction of these variables (other than alpha diversity) with the microbiome in such a way as to confound the microbiome-encoded MK-MMSE interaction. Further analyses are required to clarify this.

A reduced linear regression analysis to examine the association between the PCoA axis 1, based on the MK biosynthesis genes, and MMSE effects adjusting for individual confounding factors that drive gut microbiota variation found that the association between MMSE and the MK genes was significant for all variables with the exception of when adjusted for age, frailty, strata and number of diagnoses. This lack of significance for these variables reflects the fact that the microbiota changes are biological age-associated and so any association between MMSE and the MK genes should be viewed in this context (**Table 2**).

**Table 1. Poisson regression tests of associations between PCoA axis 1 and no. of MMSE errors after adjusting for strata and other confounding factors.**

| **Variable** | **e^RC^** | **95% CI** | ***P*-value** |
| --- | --- | --- | --- |
|  |  |  |  |
| PCoA axis 1 | 2.31 | 0.69, 7.6 | 0.17 |
| Strata (Day hospital) (n = 9) | 1.07 | 0.69, 1.61 | 0.76 |
| Strata (Longstay) (n = 17) | 4.31 | 3.26, 5.71 | <0.001 |
| Strata (Rehab) (n = 7) | 3.03 | 2.18, 4.17 | <0.001 |
| Age (years) | 1.02 | 1, 1.03 | 0.014 |
|  |  |  |  |
| PCoA axis 1 | 3.35 | 1.04, 10.71 | 0.042 |
| Strata (Day hospital) (n = 9) | 1.21 | 0.78, 1.81 | 0.38 |
| Strata (Longstay) (n = 17) | 5.07 | 3.98, 6.49 | <0.001 |
| Strata (Rehab) (n = 7) | 3.21 | 2.3, 4.45 | <0.001 |
| Gender (Male) (n = 23) | 0.84 | 0.67, 1.04 | 0.11 |
|  |  |  |  |
| PCoA axis 1 | 2.93 | 0.9, 9.45 | 0.073 |
| Strata (Day hospital) (n = 9) | 1.2 | 0.78, 1.8 | 0.39 |
| Strata (Longstay) (n = 17) | 5.09 | 4, 6.52 | <0.001 |
| Strata (Rehab) (n = 7) | 3.04 | 2.19, 4.19 | <0.001 |
| Presence of hypertension (n = 23) | 0.89 | 0.71, 1.1 | 0.28 |
|  |  |  |  |
| PCoA axis 1 | 2.78 | 0.83, 9.14 | 0.094 |
| Strata (Day hospital) (n = 9) | 1.12 | 0.73, 1.68 | 0.58 |
| Strata (Longstay) (n = 17) | 4.84 | 3.79, 6.21 | <0.001 |
| Strata (Rehab) (n = 7) | 2.91 | 2.08, 4.04 | <0.001 |
| Hypertension medication (1 medication) (n = 8) | 0.83 | 0.6, 1.11 | 0.22 |
| Hypertension medication (2 medication) (n = 6) | 0.74 | 0.46, 1.13 | 0.19 |
| Hypertension medication (3 medication) (n = 3) | 0.23 | 0.04, 0.73 | 0.041 |
|  |  |  |  |
| PCoA axis 1 | 3.14 | 0.96, 10.15 | 0.057 |
| Strata (Day hospital) (n = 9) | 1.18 | 0.76, 1.76 | 0.45 |
| Strata (Longstay) (n = 17) | 5.13 | 4.03, 6.56 | <0.001 |
| Strata (Rehab) (n = 7) | 3.06 | 2.2, 4.22 | <0.001 |
| Presence of diabetes (n = 5) | 1.04 | 0.67, 1.53 | 0.86 |
|  |  |  |  |
| PCoA axis 1 | 2.26 | 0.68, 7.45 | 0.18 |
| Strata (Day hospital) (n = 9) | 1.19 | 0.77, 1.78 | 0.42 |
| Strata (Longstay) (n = 17) | 5.42 | 4.23, 7 | <0.001 |
| Strata (Rehab) (n = 7) | 3.07 | 2.2, 4.24 | <0.001 |
| Diabetes medication (1 medication) (n = 2) | 0.43 | 0.18, 0.85 | 0.03 |
| Diabetes medication (2 medication) (n = 2) | 1.31 | 0.55, 2.62 | 0.49 |
|  |  |  |  |
| PCoA axis 1 | 3.29 | 1.01, 10.59 | 0.047 |
| Strata (Day hospital) (n = 9) | 1.12 | 0.72, 1.68 | 0.59 |
| Strata (Longstay) (n = 17) | 4.92 | 3.85, 6.31 | <0.001 |
| Strata (Rehab) (n = 7) | 2.9 | 2.07, 4.01 | <0.001 |
| Serum PK (nmol/l) | 0.79 | 0.58, 1.03 | 0.1 |
|  |  |  |  |
| PCoA axis 1 | 2.95 | 0.78, 10.93 | 0.11 |
| Strata (Day hospital) (n = 9) | 0.97 | 0.61, 1.49 | 0.9 |
| Strata (Longstay) (n = 17) | 4.94 | 3.87, 6.35 | <0.001 |
| Strata (Rehab) (n = 7) | 3.06 | 2.2, 4.21 | <0.001 |
| TNF-alpha (IU) | 1.03 | 1.01, 1.04 | <0.001 |
|  |  |  |  |
| PCoA axis 1 | 4.66 | 1.23, 17.24 | 0.022 |
| Strata (Day hospital) (n = 9) | 0.96 | 0.61, 1.45 | 0.84 |
| Strata (Longstay) (n = 17) | 4.53 | 3.53, 5.85 | <0.001 |
| Strata (Rehab) (n = 7) | 2.51 | 1.78, 3.5 | <0.001 |
| IL-6 (IU) | 1.01 | 1, 1.01 | <0.001 |
|  |  |  |  |
| PCoA axis 1 | 4.83 | 1.34, 17.12 | 0.015 |
| Strata (Day hospital) (n = 9) | 1.17 | 0.76, 1.75 | 0.46 |
| Strata (Longstay) (n = 17) | 5.14 | 3.98, 6.67 | <0.001 |
| Strata (Rehab) (n = 7) | 3.13 | 2.22, 4.36 | <0.001 |
| IL-8 (IU) | 1 | 0.99, 1 | 0.54 |
|  |  | , |  |
| PCoA axis 1 | 0.95 | 0.21, 4.28 | 0.94 |
| Strata (Day hospital) (n = 9) | 1.55 | 0.95, 2.4 | 0.063 |
| Strata (Longstay) (n = 17) | 4.72 | 3.67, 6.11 | <0.001 |
| Strata (Rehab) (n = 7) | 3.23 | 2.3, 4.48 | <0.001 |
| Shannon diversity | 0.8 | 0.61, 1.04 | 0.092 |
|  |  |  |  |
| PCoA axis 1 | 4.32 | 1.28, 14.44 | 0.018 |
| Strata (Day hospital) (n = 9) | 1.11 | 0.72, 1.66 | 0.62 |
| Strata (Longstay) (n = 17) | 4.53 | 3.53, 5.83 | <0.001 |
| Strata (Rehab) (n = 7) | 3.14 | 2.25, 4.33 | <0.001 |
| BMI (kg/m^2^) | 0.97 | 0.95, 0.99 | <0.001 |
|  |  |  |  |
| PCoA axis 1 | 3.73 | 1.07, 12.8 | 0.038 |
| Strata (Day hospital) (n = 9) | 1.07 | 0.69, 1.6 | 0.76 |
| Strata (Longstay) (n = 17) | 2.12 | 1.31, 3.38 | 0.002 |
| Strata (Rehab) (n = 7) | 1.72 | 1.13, 2.58 | 0.01 |
| Barthel Score | 0.94 | 0.91, 0.97 | <0.001 |
|  |  |  |  |
| PCoA axis 1 | 2.41 | 0.59, 9.7 | 0.22 |
| Strata (Day hospital) (n = 9) | 1.07 | 0.69, 1.6 | 0.77 |
| Strata (Longstay) (n = 17) | 4.29 | 3.24, 5.7 | <0.001 |
| Strata (Rehab) (n = 7) | 2.5 | 1.74, 3.56 | <0.001 |
| Number of diagnoses | 1.07 | 1.02, 1.13 | 0.01 |
|  |  |  |  |
| PCoA axis 1 | 4.25 | 1.26, 14.2 | 0.019 |
| Strata (Day hospital) (n = 9) | 1.15 | 0.74, 1.72 | 0.52 |
| Strata (Longstay) (n = 17) | 4.58 | 3.51, 6.01 | <0.001 |
| Strata (Rehab) (n = 7) | 2.57 | 1.76, 3.7 | <0.001 |
| Number of drugs | 1.03 | 1, 1.07 | 0.052 |
|  |  |  |  |
| PCoA axis 1 | 5.53 | 1.62, 18.56 | 0.006 |
| Strata (Day hospital) (n = 9) | 1.21 | 0.78, 1.81 | 0.38 |
| Strata (Longstay) (n = 17) | 4.76 | 3.68, 6.18 | <0.001 |
| Strata (Rehab) (n = 7) | 2.89 | 2.07, 3.99 | <0.001 |
| Antidepressants /antipsychotics (n = 20) | 1.21 | 0.98, 1.5 | 0.084 |

*e^RC^ exponentiated regression coefficient; CI confidence interval.*

**Table 2. A regression analyses to test the association between PCoA axis 1 and MMSE score adjusting for a single confounding factor.**

|  | **Estimate** | **Std. Error** | **t value** | **Pr(>\|t\|)** |
| --- | --- | --- | --- | --- |
|  |  |  |  |  |
| MMSE score | -0.003 | 0.001 | -1.912 | 0.060 |
| Age (years) | 0.002 | 0.001 | 1.906 | 0.061 |
|  |  |  |  |  |
| MMSE score | -0.002 | 0.002 | -1.173 | 0.245 |
| Strata (Day hospital) (n = 9) | 0.017 | 0.026 | 0.659 | 0.512 |
| Strata (Longstay) (n = 17) | 0.041 | 0.030 | 1.342 | 0.184 |
| Strata (Rehab) (n = 7) | 0.000 | 0.031 | -0.005 | 0.996 |
|  |  |  |  |  |
| MMSE score | -0.004 | 0.001 | -3.206 | 0.002 |
| Gender (Male) (n = 23) | 0.018 | 0.018 | 1.019 | 0.312 |
|  |  |  |  |  |
| MMSE score | -0.004 | 0.001 | -3.192 | 0.002 |
| Presence of hypertension (n = 23) | 0.013 | 0.018 | 0.750 | 0.456 |
|  |  |  |  |  |
| MMSE score | -0.004 | 0.001 | -3.128 | 0.003 |
| Hypertension medication (1 medication) (n = 8) | 0.013 | 0.027 | 0.463 | 0.645 |
| Hypertension medication (2 medication) (n = 6) | 0.025 | 0.031 | 0.818 | 0.416 |
| Hypertension medication (3 medication) (n = 3) | 0.006 | 0.043 | 0.130 | 0.897 |
|  |  |  |  |  |
| MMSE score | -0.004 | 0.001 | -3.137 | 0.002 |
| Presence of diabetes (n = 5) | 0.019 | 0.033 | 0.567 | 0.572 |
|  |  |  |  |  |
| MMSE score | -0.004 | 0.001 | -3.108 | 0.003 |
| Diabetes medication (1 medication) (n = 2) | -0.022 | 0.051 | -0.427 | 0.671 |
| Diabetes medication (2 medication) (n = 2) | 0.035 | 0.051 | 0.672 | 0.504 |
|  |  |  |  |  |
| MMSE score | -0.004 | 0.001 | -2.936 | 0.004 |
| Serum PK (nmol/l) | -0.015 | 0.017 | -0.902 | 0.370 |
|  |  |  |  |  |
| MMSE score | -0.003 | 0.001 | -2.394 | 0.019 |
| TNF-alpha (IU) | 0.003 | 0.001 | 2.141 | 0.036 |
|  |  |  |  |  |
| MMSE score | -0.003 | 0.001 | -2.422 | 0.018 |
| IL-6 (IU) | 0.000 | 0.000 | 0.352 | 0.726 |
|  |  |  |  |  |
| MMSE score | -0.004 | 0.001 | -2.740 | 0.008 |
| IL-8 (IU) | 0.000 | 0.000 | 0.011 | 0.992 |
|  |  |  |  |  |
| MMSE score | -0.001 | 0.001 | -1.131 | 0.262 |
| Shannon diversity | -0.097 | 0.017 | -5.672 | <0.001 |
|  |  |  |  |  |
| MMSE score | -0.004 | 0.001 | -3.252 | 0.002 |
| BMI (kg/m^2^) | 0.001 | 0.001 | 0.585 | 0.561 |
|  |  |  |  |  |
| MMSE score | -0.003 | 0.002 | -1.495 | 0.139 |
| Barthel Score | -0.001 | 0.002 | -0.504 | 0.616 |
|  |  |  |  |  |
| MMSE score | -0.001 | 0.001 | -0.626 | 0.533 |
| Number of diagnoses | 0.015 | 0.004 | 4.037 | <0.001 |
|  |  |  |  |  |
| MMSE score | -0.004 | 0.001 | -2.964 | 0.004 |
| Number of drugs | -0.001 | 0.003 | -0.209 | 0.835 |
|  |  |  |  |  |
| MMSE score | -0.004 | 0.001 | -2.740 | 0.008 |
| Antidepressants /antipsychotics (n = 20) | 0.010 | 0.020 | 0.528 | 0.599 |
