# Supplementary material for: Exploratory analysis of covariation of microbiota-derived vitamin K and cognition in older adults
Source: Am J Clin Nutr. 2019 Sep 13;110(6):1404–15. doi: 10.1093/ajcn/nqz220 (PMC6885478; doi:10.1093/ajcn/nqz220)
Supplement: nqz220_Supplemental_Files [file nqz220_supplemental_files.zip › McCann_etal_Supplemental_Figs.pdf]

Online Supporting Material

Exploratory analysis of covariation of microbiota-derived vitamin K and cognition in older adults

Angela McCann, Ian B. Jeffery, Bouchra Ouliass, Guylaine Ferland, Xueyen Fu, Sarah L. Booth, Tam T. T. Tran, Paul W. O'Toole, Eibhlís M. O' Connor

Supplemental Figures:

Supplemental Figure 1. Subject flowchart detailing the selection of participants in the current study.

Supplemental Figure 2. Pathway of genes involved in menaquinone biosynthesis.

Supplemental Figure 3. Results of Calinski-Harabasz index revealing four as the optimal number of clusters.

Supplemental Figure 4. NMF clustering of Vitamin K abundance based on log10 transformation of raw counts.

Supplemental Figure 5. Principal component analysis (**A**) and NMF (**B**) based on log10 normalised concentrations of 10 MK isoforms from faecal samples.

Supplemental Figure 6. Principal components analysis (PCA) and heatmap of all confounding factors.

Supplemental Figure 7. Redundancy Analysis (RDA) and correlation network based on MK isoforms.

Supplemental Figure 8. Boxplots showing the Shannon diversity of samples within the vitamin K abundance clusters.

Supplemental Figure 9. Boxplots showing the predicted gene numbers for samples within each of the vitamin K abundance clusters.

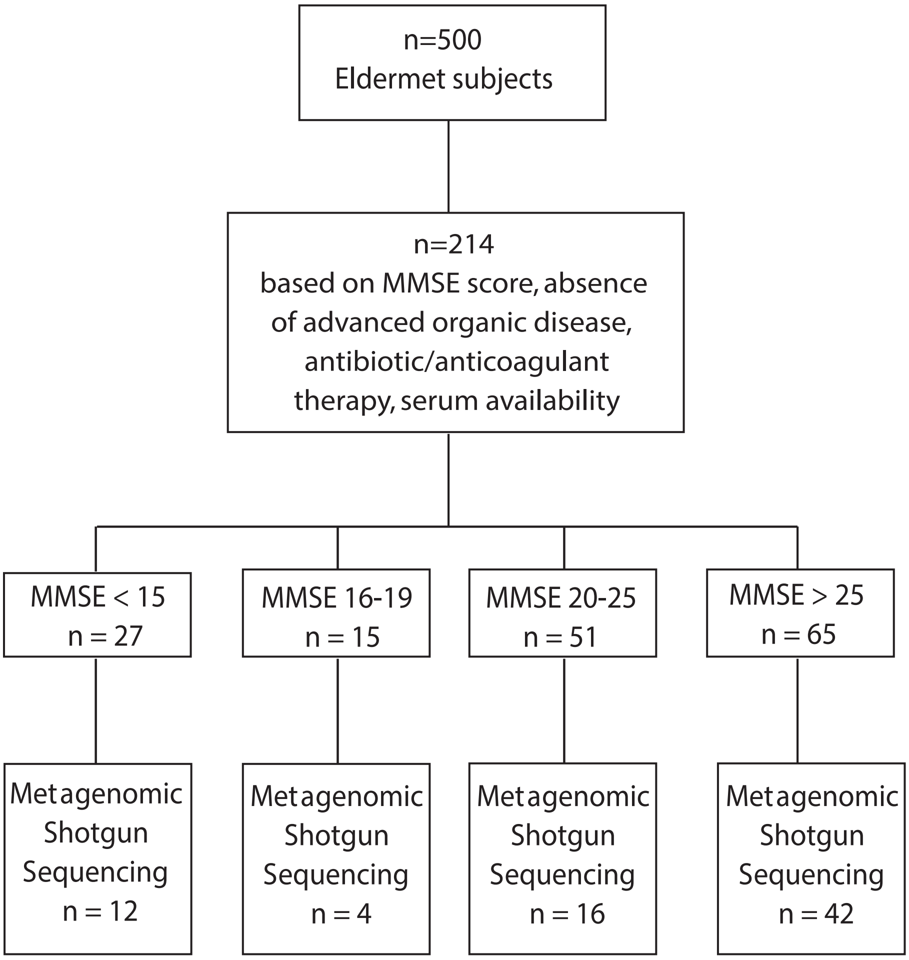

Supplemental Figure 1. Subject flowchart detailing the selection of participants in the current study.

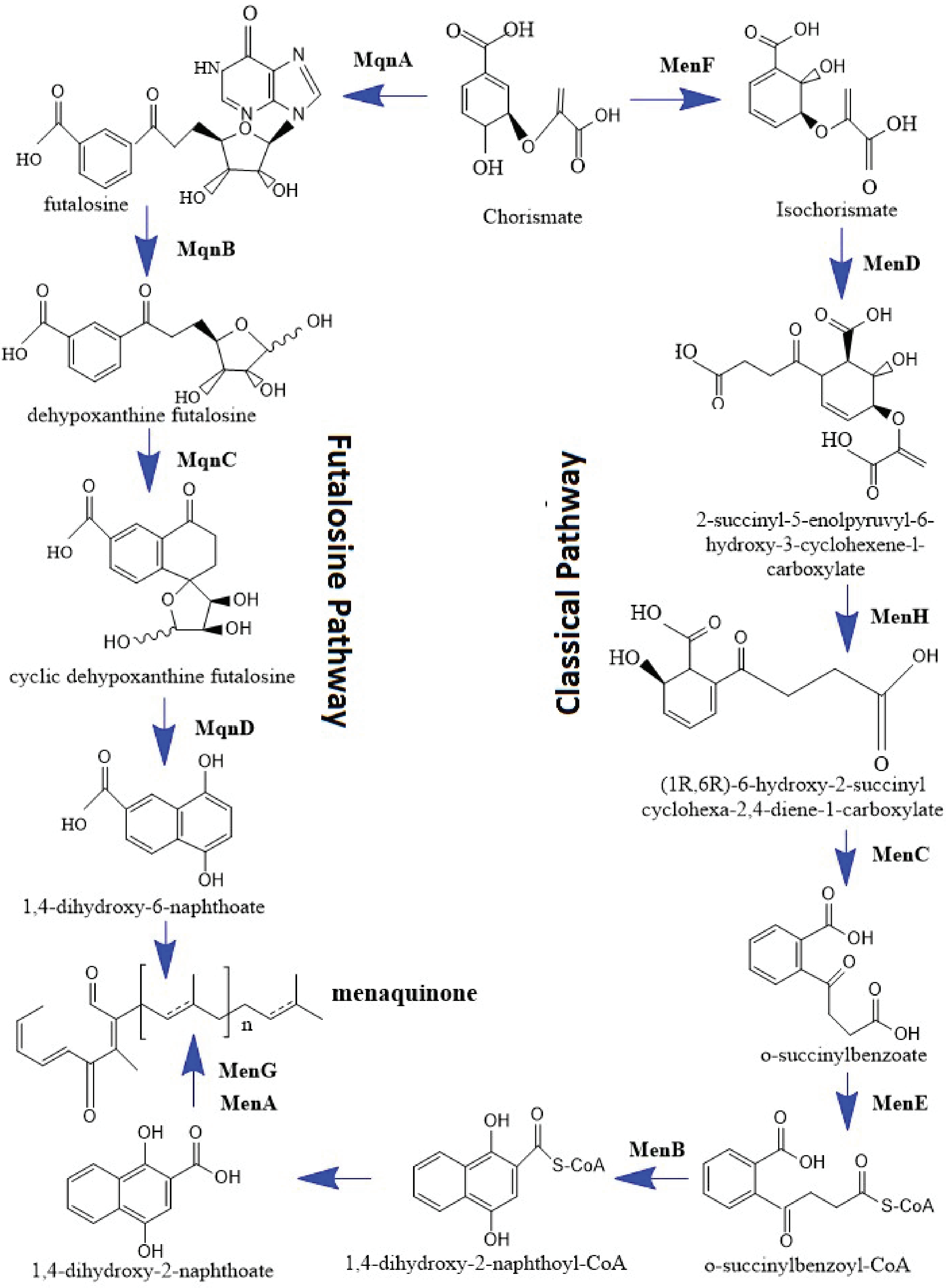

Supplemental Figure 2. Pathway of genes involved in menaquinone biosynthesis.

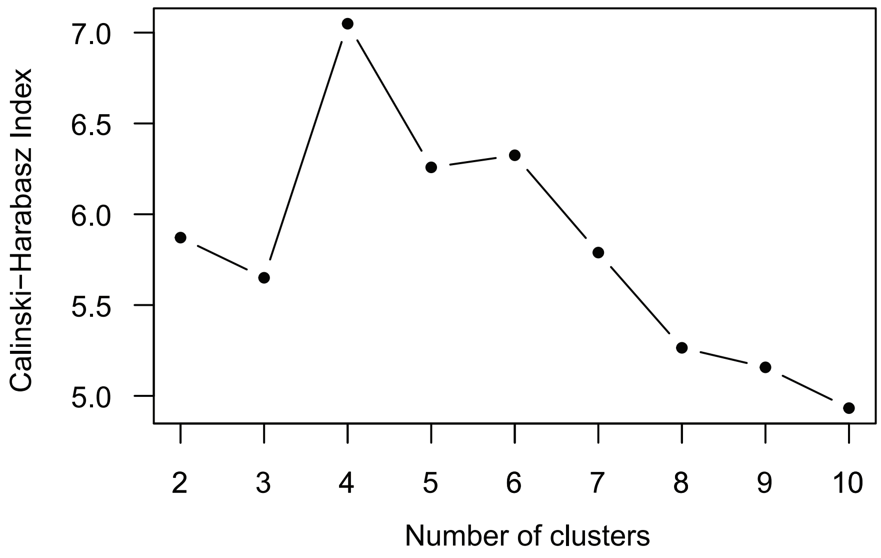

Supplemental Figure 3. Results of Calinski-Harabasz index revealing four as the optimal number of clusters.

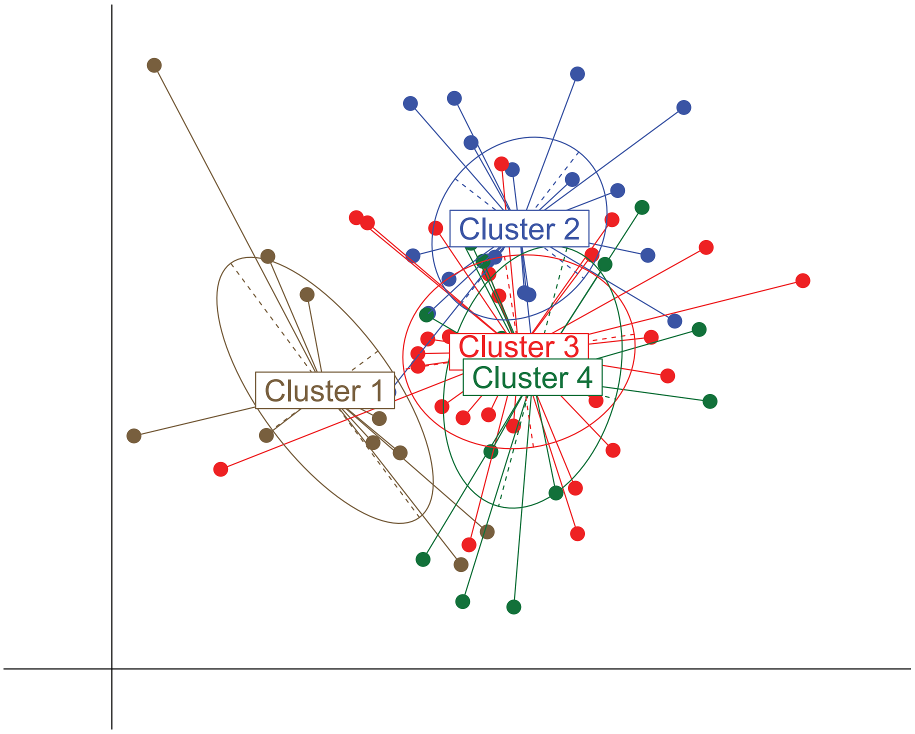

Supplemental Figure 4. NMF clustering of Vitamin K abundance based on log10 transformation of raw counts.

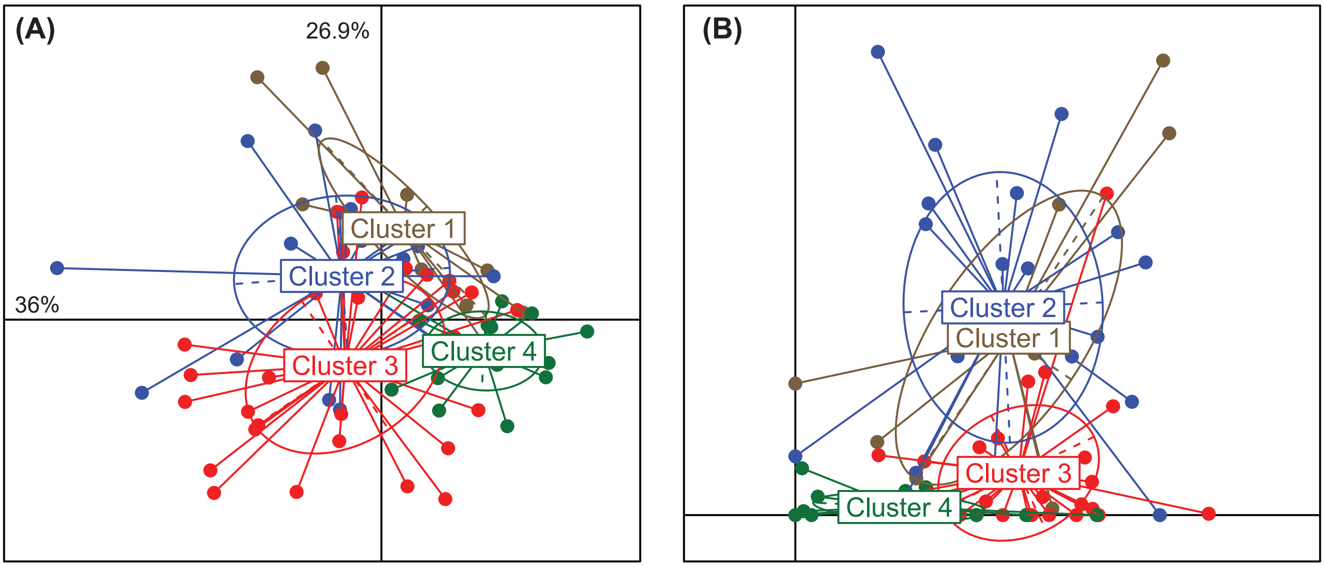

Supplemental Figure 5. Principal component analysis (**A**) and NMF (**B**) based on log10 normalised concentrations of 10 MK isoforms from faecal samples.

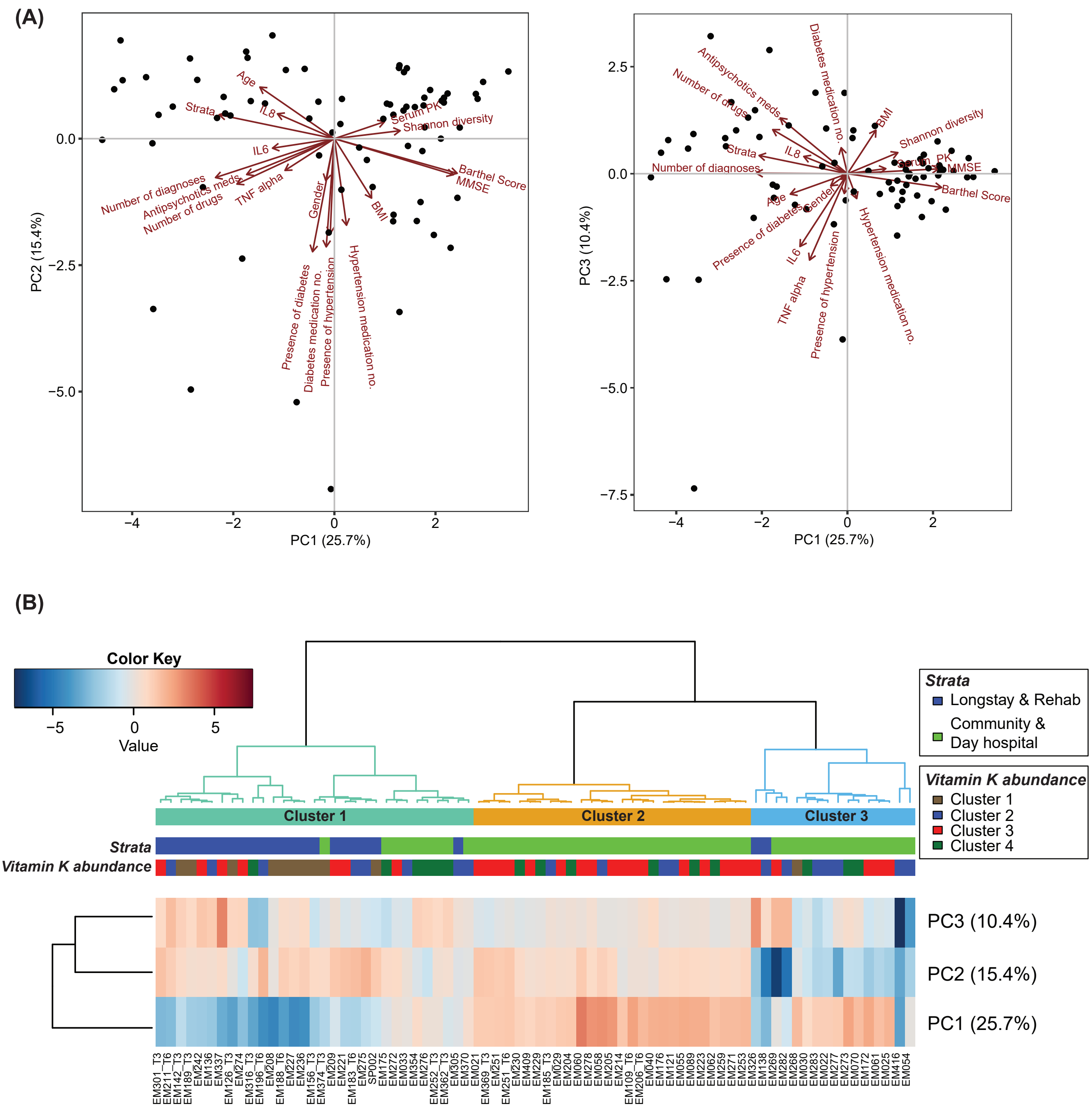

Supplemental Figure 6. Principal components analysis (PCA) and heatmap of all confounding factors. (**A**) Principal components analysis of all confounding factors from 74 elderly individuals with different cognitive ability levels. Red arrows show the direction and length - indicating the variable trend and strength of each confounding factor. PC1 correlates well with the change in frailty and comorbidity as measured by age, strata, IL-8, IL-6, number of diagnoses, number of drugs, antipsychotics medication, Barthel score, MMSE score, alpha diversity as well as serum PK; whereas hypertension, diabetes, BMI, and gender are projected along with PC2 and PC3. (**B**) Heatmap and hierarchical clustering analysis reveal three distinct clusters based on the three first axes from the PCA of the confounding factors. The coloured bars above the heatmap indicate the clusters by the confounding factors, strata or vitamin K abundance clusters. Each column represents an individual subject and each row represents a PCA axis.

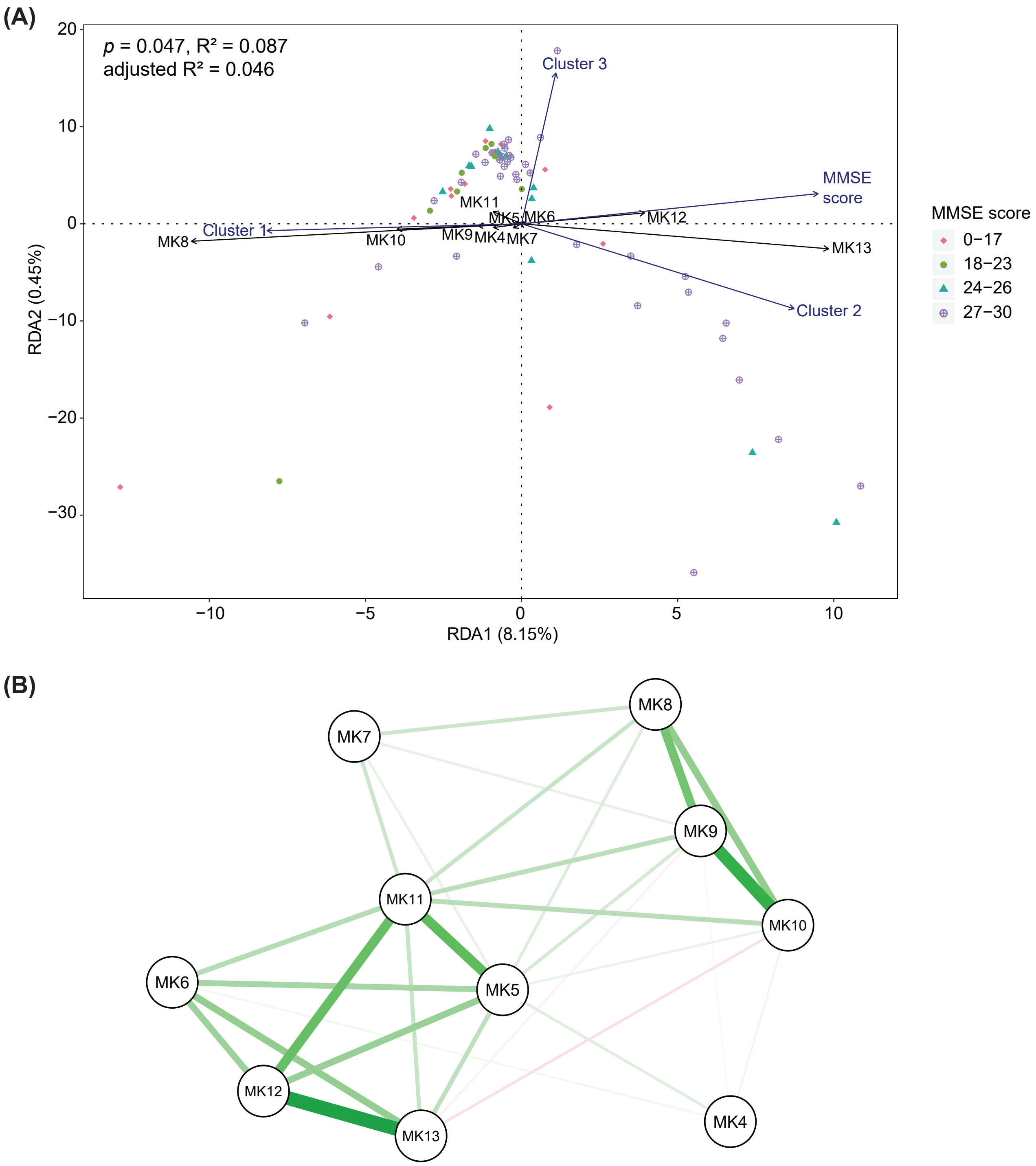

Supplemental Figure 7. Redundancy Analysis (RDA) and correlation network based on MK isoforms. (**A**) RDA of the association between MK isoforms and MMSE score, adjusted for the effects of the confounding factors. Black arrows represent MK isoforms, and blue arrows represent clusters of confounding factors and MMSE score. Samples are labelled according to MMSE score. P-value of RDA model was calculated using an analysis of variance (ANOVA). (**B**) Correlation network between MK isoforms computed based on Spearman's rank correlation coefficient. Green and red lines indicate positive and negative correlations. Line width represents the strength of the correlation. Only links with correlation coefficients value greater than 0.25 are displayed.

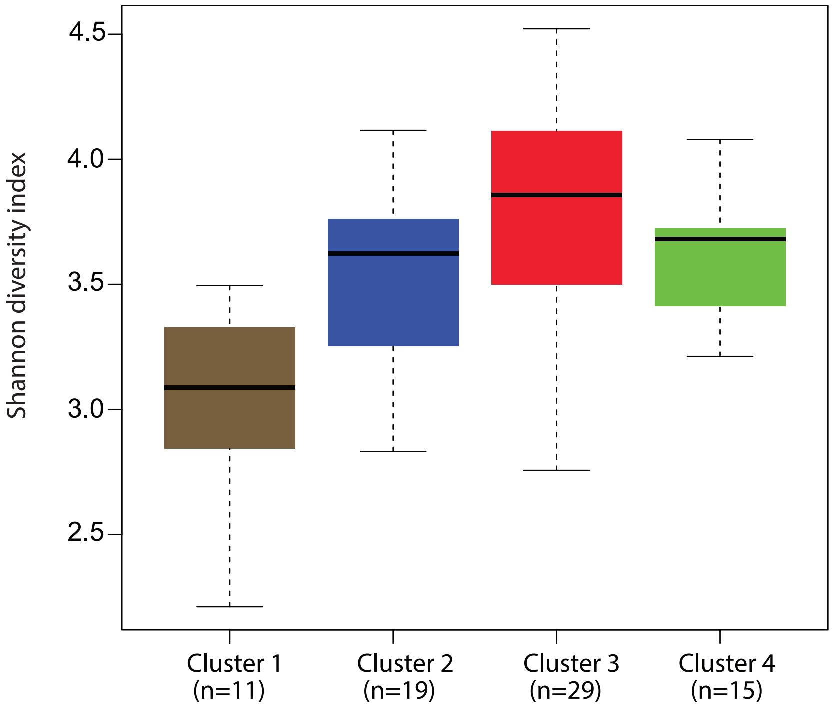

Supplemental Figure 8. Boxplots showing the Shannon diversity of samples within the vitamin K abundance clusters.

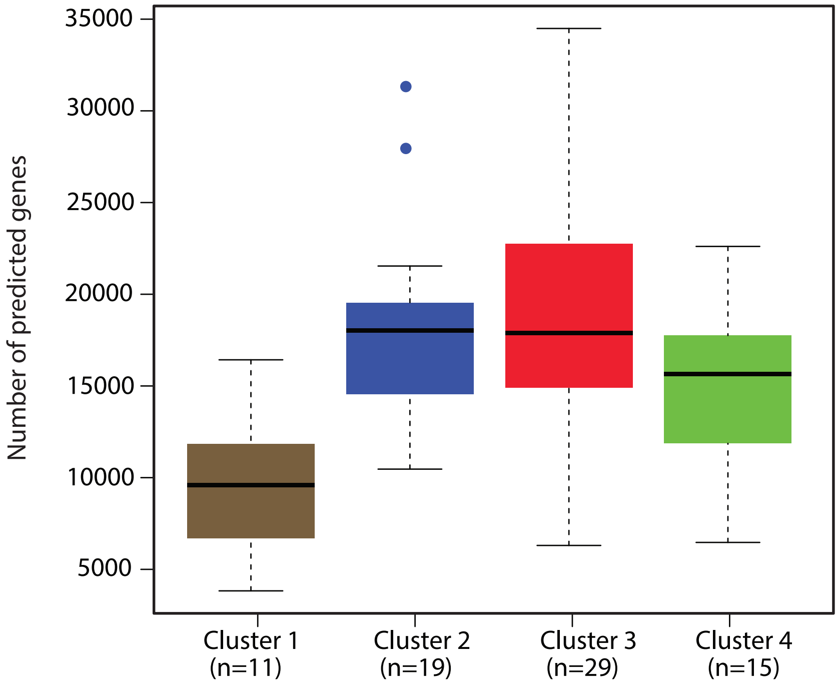

Supplemental Figure 9. Boxplots showing the predicted gene numbers for samples within each of the vitamin K abundance clusters.
